# Ribosomal protein L10 is encoded in the mitochondrial genome of many land plants and green algae

**DOI:** 10.1186/1471-2148-9-265

**Published:** 2009-11-16

**Authors:** Jeffrey P Mower, Linda Bonen

**Affiliations:** 1 Center for Plant Science Innovation and Department of Agronomy and Horticulture, University of Nebraska, Lincoln, NE, USA; 2 Department of Biology, University of Ottawa, Ottawa, Canada

## Abstract

**Background:**

The mitochondrial genomes of plants generally encode 30-40 identified protein-coding genes and a large number of lineage-specific ORFs. The lack of wide conservation for most ORFs suggests they are unlikely to be functional. However, an ORF, termed *orf-bryo1*, was recently found to be conserved among bryophytes suggesting that it might indeed encode a functional mitochondrial protein.

**Results:**

From a broad survey of land plants, we have found that the *orf-bryo1 *gene is also conserved in the mitochondria of vascular plants and charophycean green algae. This gene is actively transcribed and RNA edited in many flowering plants. Comparative sequence analysis and distribution of editing suggests that it encodes ribosomal protein L10 of the large subunit of the ribosome. In several lineages, such as crucifers and grasses, where the *rpl10 *gene has been lost from the mitochondrion, we suggest that a copy of the nucleus-encoded chloroplast-derived *rpl10 *gene may serve as a functional replacement.

**Conclusion:**

Despite the fact that there are now over 20 mitochondrial genome sequences for land plants and green algae, this gene has remained unidentified and largely undetected until now because of the unlikely coincidence that most of the earlier sequences were from the few lineages that lack the intact gene. These results illustrate the power of comparative sequencing to identify novel genomic features.

## Background

The mitochondrial proteome consists of at least 1000 different proteins. The genes encoding many of these proteins were initially encoded within the original respiring endosymbiont but have undergone intracellular transfer to the nucleus over evolutionary time, so that the proteins must be targeted back to the mitochondrion to perform their function. The number of retained mitochondrial protein-coding genes varies widely among eukaryotes, from 67 in the jakobid *Reclinomonas americana *[1] to only 3 in apicomplexans such as *Plasmodium falciparum *[2]. Genes retained in the mitochondrion encode proteins involved in fundamental mitochondrial processes such as electron transport, ATP synthesis, gene expression, and protein maturation/import. In *Reclinomonas *mitochondria, genes for the translational machinery comprise the largest single category, with 27 ribosomal protein genes [1].

In streptophytes (vascular plants, bryophytes, and charophycean green algae), the mitochondrial genome typically contains about 30 to 40 protein-coding genes of identified function. Approximately 20 of these genes are universally present, whereas the others (or a subset thereof) have been lost from various plant groups [3]. Genes encoding ribosomal proteins and subunits of the succinate dehydrogenase complex are most commonly absent [3], although loss or pseudogenization of other genes, such as *cox2 *[4,5], *nad7 *[6,7], *atp8 *[7], and cytochrome c biogenesis subunits [7,8] has occurred as well. Typically, a gene is deleted from the plant mitochondrial genome only after successful transfer of a copy to the nucleus, although examples exist where loss is correlated with functional replacement of a "native" mitochondrial ribosomal protein by a nucleus-encoded plastid or cytosolic homolog [9,10]. The timing of migration of mitochondrial ribosomal protein genes to the nucleus during eukaryotic evolution can be followed by comparative analysis [11,12].

The mitochondrial genomes of seed plants are particularly large and recombinogenic. They contain many potential unknown open reading frames (ORFs) which have often been annotated as such in genomic sequencing projects when longer than 100 codons. However, most of these ORFs are not broadly conserved, which has brought into question their potential functionality. Moreover, it is not uncommon for plant mitochondrial DNA rearrangements to give rise to novel chimeric ORFs in specific lineages, and in certain instances such ORFs are correlated with mitochondrial dysfunction in the form of cytoplasmic male sterility [13]. On the other hand, a few ORFs have shown conservation among plants, and over recent years these have been upgraded to known mitochondrial genes. This list includes *atp4 *[14,15], *atp8 *[15-17] and *mttB *(or *tatC*) [18,19], which previously were denoted as *orf25*, *orfB*, and *orfX*, respectively. Within the three complete non-vascular plant mitochondrial genomes, there is another unidentified conserved ORF, named *orf-bryo1 *in the hornwort *Megaceros aenigmaticus *[7], *orf187 *in the moss *Physcomitrella patens *[20], and *orf168 *in the liverwort *Marchantia polymorpha *[21], suggesting that it may in fact code for a functional mitochondrial product in plants.

## Results and Discussion

### Mitochondrial *orf-bryo1 *is conserved across streptophytes

To determine whether this bryophyte mitochondrial ORF might be more widespread among plants, blastp searches were performed using these three protein sequences to query the NCBI protein database. A homolog was found in the completely sequenced mitochondrial genomes of the angiosperms *Nicotiana tabacum *(*orf159b*) [22] and *Vitis vinifera *(*orf159*) [23] and, albeit with low sequence similarity, in the charophytes *Chaetosphaeridium globosum *(*orf126*) [8] and *Chlorokybus atmophyticus *(*orf295*) [24]. An unnamed predicted protein from cDNA analysis (XP_002332837) was also identified from *Populus trichocarpa*. Interestingly, the moss *orf187 *shows weak similarity to ribosomal protein L10 from several bacteria, including *Rickettsia prowazekii *and other members of the alpha-proteobacteria, the lineage from which mitochondria originated [25], as well as to mitochondrial L10 from the jakobid *Reclinomonas americana*, a protist that possesses the most "primitive" and gene-rich of all mitochondrial genomes [1]. These observations suggested that the moss *orf187 *(and its homologs) might encode mitochondrial L10 in plants. Indeed, annotated L10 domains can be found in the GenPept records for *Physcomitrella orf187 * (BAE93086) and *Chlorokybus orf295 *(ABO15139).

**Figure 1:**
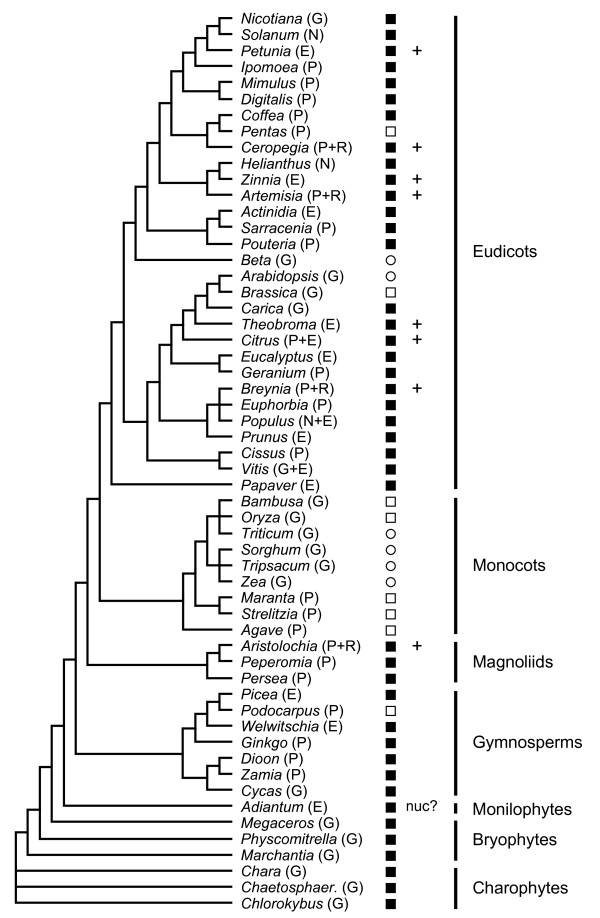
**Distribution of mitochondrial *rpl10*-like sequences in streptophytes**. Functional genes, pseudogenes, and genes lost from the mitochondrion are shown as filled squares, open squares, and open circles, respectively. Genes with evidence for expression as determined by RNA editing status are marked with a plus symbol. The 'nuc?' note next to the *Adiantum *sequence indicates that it may be encoded in the nucleus. The origin of each sequence is given in parentheses using the following abbreviations: E - EST sequence from GenBank; G - genome sequence from GenBank; N - nucleotide sequence from GenBank; P - PCR product generated during this study; R - RT-PCR product generated during this study. Phylogenetic relationships are taken from the Angiosperm Phylogeny Website [55].

A variety of computational and experimental approaches were used to determine the distribution of mitochondrial *rpl10*-like sequences among streptophytes, and the results are summarized in Figure 1. To extend the database search, tblastn queries were conducted against the nucleotide nr and EST-others databases at GenBank. Indeed, homologous unannotated ORFs are present within the complete mitochondrial genomes of the charophyte *Chara vulgaris*, [26], the gymnosperm *Cycas taitungensis *[27], and the angiosperm *Carica papaya * (EU431224) as well as in partial mitochondrial genome entries for the angiosperms *Solanum lycopersicum *and *Helianthus annuus*. In addition, several truncated and/or frameshifted sequences were identified in the mitochondrial genomes of *Brassica napus, Oryza sativa*, and *Bambusa oldhamii*, suggestive of recent erosion of the *rpl10*-like gene. Searches of the EST-others database also revealed numerous homologs from a wide range of angiosperms as well as two gymnosperms, *Picea glauca *and *Welwitschia mirabilis*. Their high nucleotide similarity to counterparts identified in completely sequenced mitochondrial genomes of other seed plants suggests that these are in fact encoded in the mitochondrial genome, unless there has been extremely recent gene transfer to the nucleus. One exception is a divergent *rpl10*-like sequence from the fern *Adiantum capillus-veneris * (DK949045) that has an amino-terminal extension of 25 residues with a weak predicted mitochondrial targeting signal, and might therefore be nuclear-located.

To determine how widely this mitochondrial *rpl10*-like gene is represented in seed plants and to gain more insight into the prevalence and timing of apparent pseudogenization in certain lineages, a PCR survey was undertaken using primers designed from the angiosperm and gymnosperm sequences identified above. Sequencing revealed the presence of this gene in another 24 seed plants, of which 5 were pseudogenes (Figure 1). Overall, these results show that homologs to the *orf-bryo1 *gene can be found across virtually all major streptophyte lineages, although it should be noted that lycophytes are not represented in this data set and no homologous sequences were detected in the mitochondrial data recently presented for *Isoetes engelmannii *[28]. Notably, the *rpl10*-like gene appears to have been independently lost at least five times during angiosperm history: from the asterid *Pentas*, from the caryophyllid *Beta*, from the crucifers *Arabidopsis *and *Brassica*, from monocots, and from the conifer *Podocarpus*.

### Angiosperm *orf-bryo1 *homologs are transcribed, edited and likely encode a functional mitochondrial L10

**Figure 2:**
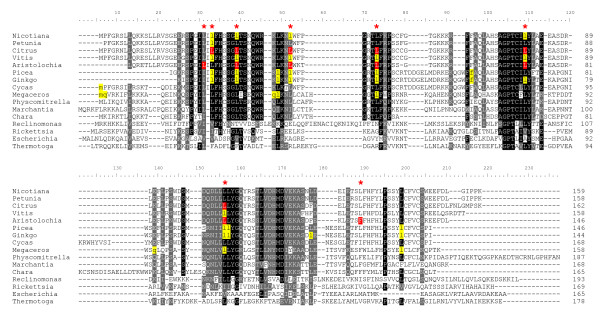
**Alignment of L10 ribosomal proteins from plant mitochondria and eubacteria**. Amino acids within a column are shaded if at least 75% are identical (black) or similar (gray). Columns in which RNA editing was observed in one or more sequences are marked with a red asterisk, and those positions are shaded in red. In the sequences translated from DNA, positions shown in lowercase and shaded in yellow were inferred to result from RNA editing by comparison to sequences from *Physcomitrella, Marchantia, and Chara *and from angiosperms with known editing data.

At the DNA level, the mitochondrial *rpl10*-like gene appears to be functional in a very wide range of streptophytes, and the derived amino acid sequence alignments for selected species are shown in Figure 2. Amino acid conservation is higher in the amino-terminal region than at the carboxy-terminus, and the latter also shows variation in length, in keeping with features also common to L10 proteins in non-plants (see below). The initiation codons for *Cycas *and *Megaceros *are predicted to be generated by C-to-U RNA editing of ACG to AUG. Within the *Megaceros *coding sequence, three potential stop codons are presumably removed by U-to-C RNA editing prior to translation, as previously postulated for many *Megaceros *mitochondrial transcripts including *orf-bryo1 *[7]. To assess whether the coding sequences are under functional constraint, the ratio (*ω*) of non-synonymous (*d*_N_) to synonymous (*d*_S_) divergence was calculated for all pairwise sequence comparisons between 6 representative streptophytes (Table 1). In all 15 cases, *ω *was less than 1 consistent with purifying selection acting to maintain the protein sequences. The average over all tests was 0.39 with a high of 0.62 between *Marchantia *and *Cycas *and a low of 0.19 between *Chara *and *Cycas*.

**Table 1:** Pairwise ω (*d*_N_/*d*_S_) for plant *rpl10 *sequences

*Petunia*						
*Citrus*	0.414					
*Cycas*	0.517	0.557				
*Physcomitrella*	0.345	0.423	0.327			
*Marchantia*	0.476	0.504	0.620	0.476		
*Chara*	0.222	0.256	0.181	0.256	0.332	
	
	*Pet.*	*Cit.*	*Cyc.*	*Phy.*	*Mar.*	*Cha.*

We have also established that the mitochondrial *rpl10*-like gene is expressed and edited in angiosperms (Table 2). The cDNA sequences obtained from four angiosperm species (*Aristolochia*, *Artemisia*, *Breynia *and *Ceropegia*) all showed C-to-U RNA editing at between 5 and 8 sites, which verifies that they were derived from RNA template rather than contaminating mitochondrial DNA. In addition, 7 edit sites were identified for *Citrus *by comparison of its EST and gene sequences. Editing in all 5 plants predominantly alters the encoded amino acids, with each coding sequence having only one silent editing event. Furthermore, these non-synonymous editing events improve protein similarity of the angiosperm sequences to one another and to species that are known to have infrequent editing, such as *Physcomitrella *[29], or no editing, as for *Marchantia *[21] and *Chara *[26]. This pattern of editing is characteristic for functional plant mitochondrial genes but not necessarily for pseudogenes [30], and most unconserved ORFs are not edited at all [31-33]. The *rpl10*-like EST sequences provided further evidence of transcription, although in the absence of accompanying DNA sequence information the evidence is less certain. The EST sequences from *Petunia, Theobroma*, and *Zinnia *generally have T at confirmed edit positions and therefore likely derive from genuine RNA rather than mitochondrial DNA contamination, although it cannot be excluded that some of these T residues are already encoded in the genome. In contrast, homologs based on EST data from additional plants lack several expected edits and reflect either mitochondrial DNA contamination or partially edited transcripts (data not shown). In total, *rpl10*-like sequences from 8 distantly-related angiosperms provide strong evidence for appropriate expression at the RNA level (Figure 1).

**Table 2:** The effect of RNA editing on amino acid sequence

		Confirmed Nonsilent Editing Positions^a^							
		
Species	Evidence	31	33	39	52	73	109	156	189
*Aristolochia*	DNA+cDNA	P>L	L	S>L	P>L	P>L	S>L	S>L	L>F
*Artemisia*	DNA+cDNA	L	S>L	S>L	P>L	L	S>L	L	L
*Breynia*^b^	DNA+cDNA	L	S>L	S>L	P>L	P>L	L	S>L	L
*Ceropegia*^b^	DNA+cDNA	L	***S***	S>L	P>L	L	S>L	L	F
*Citrus*	DNA+EST	L	S>L	S>L	S>L	P>L	S>L	S>L	L
									
*Petunia*	EST	L	***S***	L	L	L	L	L	L
*Theobroma*	EST	L	L	L	L	L	L	L	L
*Zinnia*	EST	L	L	L	L	L	L	L	***S***
									
*Cycas*	DNA	L	L	L	L	L	L	L	I
*Physcomitrella*	DNA	L	L	L	L	L	L	L	F
*Marchantia*	DNA	L	L	L	I	L	L	L	F
*Chara*	DNA	I	L	L	L	L	L	L	F

a: Non-conserved amino acids are shown in bold italic type.

b: Two editing events occur at codon position 52 for *Breynia *and *Ceropegia*.

In Figure 2, the amino acid alignment of plant and charophycean green algal mitochondrial *orf-bryo1 *homologs also includes the *Reclinomonas americana *mitochondrion-encoded L10 protein and homologs from the eubacteria *Escherichia coli*, *Rickettsia prowazekii*, and *Thermotoga maritima*. The L10 ribosomal protein is universally present in the ribosomes of eubacteria, archaea, and eukaryotes, and the crystal structure of L10-L7/L12 stalk has been determined [34]. It is worth noting that the amino-terminal domain is more highly conserved than the carboxy-terminal half. For example, the *Rickettsia prowazekii *and *E. coli *L10 proteins share only about 26% amino acid identity over their full length, whereas the beta-1 to alpha-5 region (of 85 amino acids) within the amino-terminal half shows ~35% identity. It is the amino-terminal domain of L10 (or more specifically, the alpha-1 to alpha-3 region) that binds directly to the large subunit ribosomal RNA, whereas the carboxy-terminal domain of L10 (and alpha-8 in particular) interacts with the L7/L12 stalk; together with L11, this complex plays a key role in recruiting translation factors to the ribosome and stimulating GTP hydrolysis [34,35]. The flowering plant mitochondrial L10 proteins share about 23% amino acid identity with the *Rickettsia *L10 homolog over the amino-terminal beta-1 to alpha-5 region of 85 amino acids, compared to 27-28% identity seen between *Rickettsia *L10 and the comparable region of the *Physcomitrella *or *Reclinomonas *mitochondrial counterparts. Of particular note are several highly conserved blocks that are believed to be important for protein structure [34]. They contain Gly (and Pro) residues for beta-turns between beta1-alpha2 and alpha4-beta3 in L10 proteins of eubacteria and archaea. Interestingly, 7 of 8 positions of RNA editing lie within conserved blocks, consistent with their functional importance, a hallmark of RNA editing in plant mitochondria [36].

**Figure 3:**
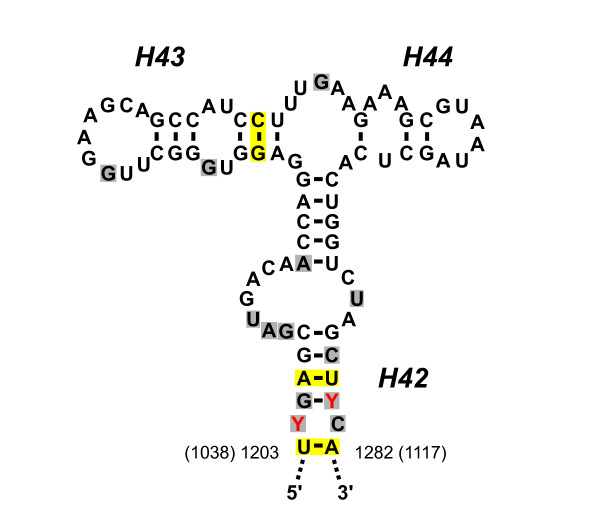
**Sequence and structure of the LSU rRNA region that binds to L10 ribosomal protein**. Shown are helices H42, H43 and H44 of the LSU rRNA. The primary sequence shown is a consensus of this mitochondrial 26S rRNA region from *Marchantia, Physcomitrella*, and numerous seed plants, with differences shown in red. Positions that differ between plant mitochondria and bacteria (represented by *E. coli*) are shaded in gray. Yellow shading indicates compensatory changes in *E. coli *that maintain base pairing in stem regions. Nucleotide coordinates are shown for *Triticum aestivum *mitochondrial 26s rRNA [56] and in parentheses for *E. coli *23s rRNA [34,35].

In bacterial, archaeal and eukaryotic cytosolic ribosomes, the amino terminal domain of the L10 protein is known to bind specifically to helices H42, H43, and H44 of the large subunit rRNA [34,35], and in plant mitochondria, this helical region of the 26S rRNA has retained the correct structure for L10 binding and is very highly conserved among streptophytes (Figure 3). Indeed this stretch of 80 nt is identical in sequence among most seed plants and there has been only one nucleotide substitution relative to either the *Physcomitrella *or *Marchantia *homolog, that is, during a period of about 400 million years. Thus, it seems likely that a conventional L10 protein (or at least for the amino-terminal portion) will be present in plant mitochondrial ribosomes.

### Status of mitochondrial L10 in grasses and crucifers

For the reasons discussed above, one might expect that all seed plants would possess a mitochondrial-type *rpl10 *gene either within the mitochondrion or alternatively within the nucleus since the simplest explanation for cases of gene loss from the mitochondrion (see Figure 1) is that successful gene transfer to the nucleus has occurred. Curiously, no mitochondrial-type L10 protein sequences were detected in tblastn searches of the completely sequenced nuclear genomes of Arabidopsis [37] or rice [38,39]. However, both these genomes do contain duplicated copies of the chloroplast-derived *rpl10 *gene (data not shown). In land plants, the chloroplast *rpl10 *gene is located in the nucleus, and proteomic analysis of spinach chloroplast ribosomes has established its precise protein content [40]. The chloroplast L10 orthologs in Arabidopsis (NP_196855) and rice (NP_001049761) share about 70% amino acid identity (excluding the acquired N-terminal targeting extensions). In contrast, the second chloroplast-type L10-related copy shows only ~41% amino acid identity between the Arabidopsis (NP_187843) and rice (NP_001054498) counterparts, and these proteins are predicted to be localized in the mitochondrion based on targeting programs such as TargetP [41], PSort [42], and Predotar [43] Interestingly, the two Arabidopsis chloroplast-derived L10 paralogs are more closely related to each other (~58% identity) than are two rice ones (~46% identity), suggesting a more recent duplication event in the crucifer lineage. This would also be consistent with their independent recruitment as functional substitutes for the mitochondrial L10 protein at different times during angiosperm evolution, although it cannot be formally excluded that gene conversion events in the Arabidopsis lineage contribute to the higher sequence similarity.

Although the duplicated chloroplast-type L10-related gene is an attractive candidate to serve as a replacement in the mitochondrial ribosome for those plants which lack the "native" mitochondrial *rpl10 *gene, these proteins in Arabidopsis and rice lack a number of the expected conserved residues, ones that are observed in the plant mitochondrion-encoded genes. Alternative possibilities are that the chloroplast L10 might be dual targeted to both the plastid and the mitochondrion or that the cytosolic ribosomal protein L10 counterpart (called L10e or P0) has been recruited. It is perhaps even possible that plants such as rice and *Brassica*, which possess what appear to be remnant pseudogene fragments in the mitochondrion, actually have several short genes (mitochondrial or nuclear) that generate a discontinuous L10 protein structure, a phenomenon observed for the mitochondrial *rpl2 *gene in certain flowering plants [44]. Finally, it is worth noting that non-homologous proteins have been known to perform molecular mimicry in the evolution of the large ribosomal subunit among eubacteria and archaea [45].

## Conclusion

In summary, these observations provide strong evidence that a functional *rpl10 *gene exists in the mitochondrion of many streptophytes. Despite the fact that there are now over 20 streptophytes with complete mitochondrial genome sequences, this gene has been missed until now due to the unlikely coincidence that most of the plant mitochondrial genomes that were first completely sequenced - the crucifers *Arabidopsis thaliana *[46] and *Brassica napus *[33]; the grasses *Oryza sativa *[32], *Zea mays *[47] and *Triticum aestivum *[48]; and the sugar beet *Beta vulgaris *[49] - are from lineages where this gene has been lost or pseudogenized. Only with the more recent sequence data from diverse streptophytes such as *Cycas taitungensis *[27], *Physcomitrella patens *[20] and *Megaceros aenigmaticus *[7] does the general pattern emerge that this gene is in fact widely present. Indeed, the bryophyte *orf-bryo1 *sequences were particularly informative in bridging the evolutionary distance between mitochondrial L10 gene homologs in seed plants and those of charophycean green algae/protists, which nicely illustrates the power of obtaining sequence information from diverse organisms in order to reconstruct events related to gene and genome history.

## Methods

Total genomic DNAs and RNAs were isolated using the DNeasy and RNeasy Plant Mini Kits (QIAGEN) from leaf tissue available in the living collection of the Beadle Center Greenhouse (University of Nebraska). To prepare first-strand cDNA, RNAs were treated with DNase I (Fermentas) to remove contaminating DNA and then reverse transcribed using M-MuLV Reverse Transcriptase (Fermentas) and random hexamers (Fermentas) according to the manufacturer's instructions.

Sequences for *rpl10 *were amplified from DNA or cDNA by polymerase chain reaction using GoTaq DNA Polymerase (Promega) and forward primer F1 (5'-ATGCCATTCGGAAGAAGTMT) with reverse primer R159 (5'-TTAGGTGGTATYCCGAGATYGA) or R148 (5'-GGAACACACGAAASAAAGATATRAAC). Each reaction was run on a DNA Engine Dyad (Bio-Rad) for 35 cycles (30 sec at 94°C, 1 min at 48°C, 2 min at 72°C), with an initial step of 3 min at 94°C and a final step of 10 min at 72°C. Amplified products were sequenced on both strands at the High-Throughput Genomics Unit (University of Washington). Sequences generated in this study were deposited in GenBank under accession numbers GQ402491-GQ402514; additional sequences used in the comparative analysis were downloaded from GenBank (Table 3).

**Table 3:** Taxonomy and GenBank accession numbers for *rpl10 *sequences in this study

Group	Order	Species	Accn. No.
Eudicots	Asterales	*Artemisia dranunculus*	GQ402491
		*Helianthus annuus*	AM183222
		*Zinnia violacea*	AU304033
	Brassicales	*Arabidopsis thaliana*	Y08501
		*Brassica napus*	AP006444
		*Carica papaya*	EU431224
	Caryophyllales	*Beta vulgaris*	BA000009
	Ericales	*Actinidia deliciosa*	FG448607
		*Pouteria sapota*	GQ402492
		*Sarracenia purpurea*	GQ402493
	Gentianales	*Ceropegia woodii*	GQ402494
		*Coffea arabica*	GQ402495
		*Pentas lanceolata*	GQ402496
	Geraniales	*Geranium sanguineum*	GQ402497
	Lamiales	*Digitalis purpurea*	GQ402498
		*Mimulus guttatus*	GQ402499
	Malpighiales	*Breynia disticha*	GQ402500
		*Euphorbia tirucalli*	GQ402501
		*Populus trichocarpa*	XM_002332800
	Malvales	*Theobroma cacao*	CU593370
	Myrtales	*Eucalyptus gunnii*	CT984759
	Ranunculales	*Papaver somniferum*	FG608946
	Rosales	*Prunus dulcis*	BU574137
	Sapindales	*Citrus sinensis*	GQ402502
	Solanales	*Ipomoea sp.*	GQ402503
		*Nicotiana tabacum*	BA000042
		*Petunia axillaris*	FN003412
		*Solanum lycopersicum*	FJ374974
	Vitales	*Cissus tuberosa*	GQ402504
		*Vitis vinifera*	FM179380
			
Monocots	Asparagales	*Agave americana*	GQ402511
	Poales	*Bambusa oldhamii*	EU365401
		*Oryza sativa*	BA000029
		*Sorghum bicolor*	DQ984518
		*Tripsacum dactyloides*	DQ984517
		*Triticum aestivum*	AP008982
		*Zea mays*	AY506529
	Zingiberales	*Maranta leuconeura*	GQ402513
		*Strelitzia reginae*	GQ402512
			
Magnoliids	Laurales	*Persea sp.*	GQ402505
	Piperales	*Aristolochia elegans*	GQ402506
		*Peperomia sp.*	GQ402507
			
Gymnosperms	Coniferales	*Picea glauca*	EX322775
		*Podocarpus macrophyllus*	GQ402514
	Cycadales	*Cycas taitungensis*	AP009381
		*Dioon edule*	GQ402508
		*Zamia pumila*	GQ402509
	Ginkgoales	*Ginkgo biloba*	GQ402510
	Gnetales	*Welwitschia mirabilis*	DT601028
			
Monilophytes	Filicales	*Adiantum capillus-veneris*	DK949045
			
Bryophytes	Dendrocerotales	*Megaceros aenigmaticus*	EU660574
	Funariales	*Physcomitrella patens*	AB251495
	Marchantiales	*Marchantia polymorpha*	M68929
			
Charophytes	Charales	*Chara vulgaris*	AY267353
	Chlorokybales	*Chlorokybus atmophyticus*	EF463011
	Coleochaetales	*Chaetosphaeridium globosum*	AF494279

Sequences were aligned using Muscle 3.7 [50] and manually adjusted in BioEdit 7.0.9 [51]. Edit sites were identified by comparison of DNA sequences with cDNA and/or EST sequences. To examine levels of functional constraint, poorly-aligned regions were first identified and removed using Gblocks 0.91b [52], then pairwise *d*_N _and *d*_S _were computed in MEGA 4.0.2 [53] using the Nei-Gojobori Model with a Jukes-Cantor correction for multiple hits.

## Note added in proof

Another group has independently discovered the *rpl10 *gene in the mitochondrial genome of plants [54]. Similar to our study, Kubo and Arimura find that the mitochondrial gene is widely distributed among plants, is transcribed and RNA edited in multiple species, and has been lost from several lineages, including *Arabidopsis *and rice. These authors suggest, as we do, that a duplicated copy of the nucleus-encoded chloroplast *rpl10 *gene has functionally replaced the lost mitochondrial *rpl10 *gene independently in *Arabidopsis *and rice. For both species, they experimentally show that these putative mitochondrially-functioning L10 proteins have targeting signals that indeed induce localization to the mitochondrion, and also the chloroplast.

## Authors' contributions

JPM conceived of the study, performed experimental and computational work, analyzed results, prepared figures and tables, and drafted the manuscript. LB also performed computational analyses, analyzed results, and helped draft the manuscript. Both authors read and approved the final manuscript.
